# Corrigendum to “Evaluation of injury threshold from the number of rib fracture for predicting pulmonary injuries in blunt chest trauma” [Heliyon 9 (4), (April 2023) Article e15278]

**DOI:** 10.1016/j.heliyon.2023.e18872

**Published:** 2023-08-02

**Authors:** Kazunori Fukushima, Masahiko Kambe, Yuto Aramaki, Yumi Ichikawa, Yuta Isshiki, Jun Nakajima, Yusuke Sawada, Kiyohiro Oshima

**Affiliations:** a Department of Emergency Medicine, Gunma University Graduate School of Medicine, Maebashi, Gunma, Japan; b ER General Medical Center, Saitama Sekishinkai Hospital, Sayama, Saitama, Japan

In the original published version of this article, the authors provided the results of the abstract incorrectly and the result of the abstract alone has been updated. *The correct version of the result in the abstract can be found below*:

Original:

"The number of rib fractures was associated with an increased risk of pulmonary injuries: pulmonary contusion (odds ratio [OR] 1.30, 95% confidence interval [CI] 1.14–1.48, p < 0.05); hemothorax (OR 1.22, 95% CI 1.08–1.38, p < 0.05); pneumothorax (OR 1.15, 95% CI 1.02–1.30, p < 0.05); and hemopneumothorax (OR 1.14, 95% CI 1.01–1.28, p < 0.05)."

Requested Change:

"The number of rib fractures was associated with an increased risk of pulmonary injury: all pulmonary injuries, OR 1.85, 95% CI 1.59–2.16, p < 0.01; pulmonary contusion, OR 1.50, 95% CI 1.33–1.69, p < 0.01; hemothorax, OR 1.49, 95% CI 1.32–1.68, p < 0.01, and pneumothorax, OR 1.47, 95% CI 1.30–1.65, p < 0.01."

The authors apologize for the errors. Both the HTML and PDF versions of the article have been updated to correct the errors.

## Declaration of interest statement

The authors declare the following conflict of interests: Fukushima, Kambe, Aramaki, and Oshima received research funding for this study from the SUBARU Corporation; however, the SUBARU Corporation had no role in study design, data collection and interpretation, or the decision to submit the work for publication. Ichikawa, Isshiki, Nakajima, and Sawada have no conflicts of interest to declare related to the study.

